# RegScan: a GWAS tool for quick estimation of allele effects on continuous traits and their combinations

**DOI:** 10.1093/bib/bbt066

**Published:** 2013-09-05

**Authors:** Toomas Haller, Mart Kals, Tõnu Esko, Reedik Mägi, Krista Fischer

**Keywords:** GWAS, genome-wide analysis, linear regression, continuous traits, combinatorial traits, metabolomics

## Abstract

Genome-wide association studies are becoming computationally more demanding with the growing amounts of data. Combinatorial traits can increase the data dimensions beyond the computational capabilities of the current tools. We addressed this issue by creating an application for quick association analysis that is ten to hundreds of times faster than the leading fast methods. Our tool (RegScan) is designed for performing basic linear regression analysis with continuous traits maximally fast on large data sets. RegScan specifically targets association analysis of combinatorial traits in metabolomics. It can both generate and analyze the combinatorial traits efficiently. RegScan is capable of analyzing any number of traits together without the need to specify each trait individually. The main goal of the article is to show that RegScan can be the preferred analytical tool when large amounts of data need to be analyzed quickly using the allele frequency test.

**Availability:** Precompiled RegScan (all major platforms), source code, user guide and examples are freely available at www.biobank.ee/regscan.

**Requirements: **Qt 4.4.3 or newer for dynamic compilations.

## INTRODUCTION

Genome-Wide Association Studies (GWAS) have successfully identified common variants of the human genome associated with the common diseases and traits. International research consortia have uncovered the effects of thousands of genetic makers in complex traits and diseases [1]. Traditionally, linear regression is used to study the association of marker frequencies and continuous traits and the *P*-value of association is the main metric for initial filtering [2]. It is becoming commonplace to study tens of thousands of individuals, tens of millions of markers and the combinations of various continuous traits (so-called combinatorial traits), mostly ratios, leading to a large number of combinations to be tested. The number of traits is especially large in the metabolomics studies where the trait ratios are considered interesting owing to their potential to shed light on metabolic pathways [3–6]. These analyses are often limited by the available computational resources. Yet, the GWAS consortia, among the others, are looking for ways to efficiently study combinatorial traits. The field is in need of a tool with a strong emphasis on speed.

We created a tool (RegScan) that considerably accelerates association studies. RegScan performs linear regression analysis (allele frequency test) and identifies the statistically significant associations between markers and traits maximally fast. Its computational speed benefit is best used in studying large numbers of combinatorial traits.

## IMPLEMENTATION

RegScan is a command line application written primarily in C++/Qt [7]. It uses the least squares approach to fit the linear regression model without introducing new modifications to the standard technique. The fast execution speed as compared with the other tools is achieved entirely by using special efficient computational techniques. In some functions, this means resorting to C-style programming instead of C++. Computational overhead is reduced as much as possible by avoiding the slower C++/Qt functions where execution speed is rate limiting and relying on Qt framework only for higher-level functions. Every function is created with the goal to minimize the number of elementary operations and choosing methods that require less time. The order of conducting individual steps is carefully considered and the fastest combinations are favored. The standard techniques of efficient programming, such as reducing time complexity, are honored wherever possible. The main speed gain is derived from an original data file reading mechanism making efficient use of C language function fread(). Reading large data from file and writing it into the file is generally the most time-consuming step. RegScan reduces the number of individual read/write events by reading data in large chunks in the binary mode and then efficiently parsing the data in the memory to retrieve the information. The parsing step is carried out by an original method that sequentially compares each byte in the stream with all integers to block-wise reconstruct the original values. Computational speed gain becomes more pronounced with the increasing number of traits because analyzing additional traits involves no additional significant read events. Care is taken to ensure that the files are read no more than once and the information that is often referred to is stored in the memory early on during runtime. The operations are always carried out with the minimal number of significant digits to save time. Approximations are used if they do not lead to sacrificing the analytical quality. Look-up tables are preferred to runtime calculations. If the value that is looked up is not present in the table, fast and accurate interpolation techniques are used to fill in the missing value. This technique is used in the *P*-value calculation. Additional methods of accelerating the analysis include setting various restrictive filters before the analysis to bypass the calculations that are not of interest to the user. These optional filters are fully controlled by the user and are explained in the RegScan User Manual.

**Figure 1:**
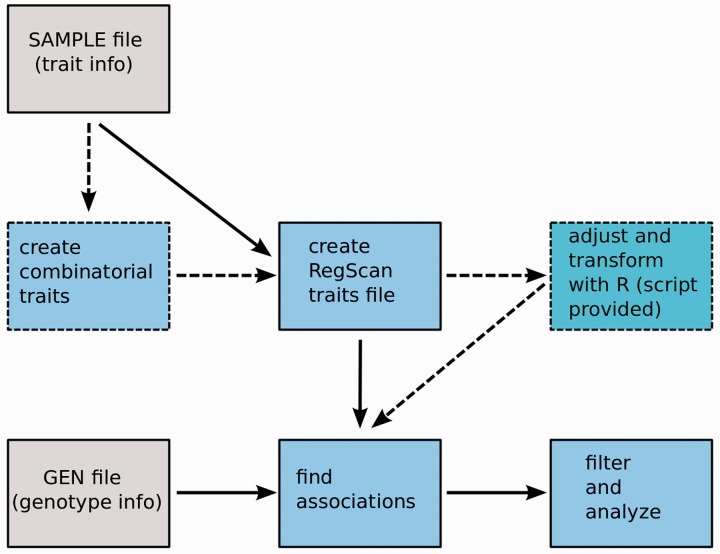
General RegScan workflow. Creating combinatorial traits and adjustments/transformations are optional (dashed boxes).

RegScan uses allele dosage to perform linear regression analysis. It works with the Oxford GEN/SAMPLE file format [8] and can also read gzip-compressed GEN files. RegScan can automatically generate all possible combinatorial traits and channel them into fast linear regression analysis. It is capable of handling missing phenotypes and has adequate error-catching mechanisms. While designed for the analysis of combinatorial traits, this is not a requirement. The general workflow of Regscan is presented in Figure 1. The analytical pipeline also uses R (script provided with RegScan) for adjustments and transformations. Effect size, *P*-value, standard error (SE) of slope and minor allele count can be used to filter the results during runtime if needed. The output can be additionally filtered and analyzed with RegScan to detect true positives or create subsets. RegScan is a collection of analytical scripts that provide functions additional to the association analysis by linear regression: (i) counting and extracting markers associated with combinatorial traits or traits associated with markers, (ii) applying unlimited number of filters to identify associations of interest or extract any subset from the results, (iii) evaluating the statistical significance of the combinatorial trait–marker associations (elaborated below). If needed, RegScan can be efficiently used to prepare condensed data sets for other tools for the types of analyses not supported by RegScan, as it is capable of quickly assessing the associations.

## SPEED TESTING

**Figure 2:**
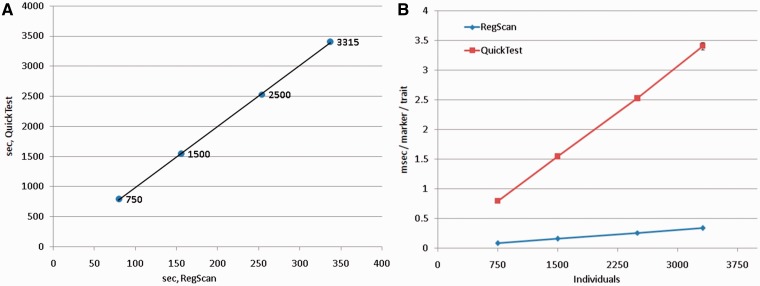
Analysis time (RegScan versus QuickTest) with 1 million markers, one trait and variable number of individuals (750–3315). (**A**) Relative speed gain of RegScan over QuickTest; slope = 10.14, (**B**) Computational speed of RegScan and QuickTest as a function of the number of individuals. A colour version of this figure is available at BIB online: http://bib.oxfordjournals.org.

SNPTEST 2.4.1 [9] and QuickTest 0.97 [10] were used as references in the tests, as these two widely used computational tools perform linear regression analysis and output all of the three commonly used statistical parameters: *P*-value, effect size (β) and SE. We conducted tests to estimate (i) the analysis speed gain of RegScan over the reference tools, (ii) computational speed as a function of data size and various settings. The results are presented in Supplementary Data. Briefly, compared with the other software packages, RegScan was always the fastest, and QuickTest the second fastest. QuickTest was therefore chosen for computational speed comparison tests. A typical RegScan analysis was 10× faster than QuickTest on a 2.3 GHz (Scientific Linux 6.3) with one trait and various numbers of individuals (Figure 2). RegScan analyzed our test data set (TDS, 38.02 million markers, 3315 individuals) and one trait in 3.4 h (0.34 ms/marker) as opposed to the QuickTest time of 36.2 h. The analysis speed per trait increased significantly when multiple traits were analyzed in one go. Experiments with 6216 traits led to computational times <10 min/trait with the TDS. The linear regression analysis proceeded at 0.011 ms/trait/marker (30× faster than with a single trait).

The computational times can be additionally shortened by allowing RegScan to allocate more Random-access memory (RAM). Further significant speed gain results from setting various restrictive filters such as a higher minor allele count level, lower SE level, etc. Combining all methods of computational speed reduction can result in analysis speed that is several orders of magnitude faster than with the other common GWAS tools.

Computational speed can be of greatest importance in certain situations such as creating large databases of associations by brute force or studying combinatorial traits. For example, a data set with only 112 metabolite concentrations will create 6160 pair-wise combinations. When analyzed individually with other tools and the TDS (see above) the time spent will be >26 processor years. Analyzing these traits individually with RegScan would take about 2.6 processor years. However, when fed into the RegScan analysis all at once, these traits will require only about one processor month, or even less when applying additional optional RegScan filters.

## QUANTITATIVE COMPARISON OF RESULTS

An allele frequency test was performed with RegScan and the reference tools on a data set of 40 765 randomly chosen markers from the 1000 Genomes reference panel and 873 individuals to quantitatively compare the results [11]. The results for *P*-value, effect size (β) and SE agreed well between all tools (Table 1). The somewhat larger, albeit minor, *P*-value differences between RegScan and the other tools originated from a different computational method used—interpolation of precomputed values—and from the rounding effects (Table 2). The tests, however, confirmed that (i) the RegScan results do not differ significantly from the other commonly used tools for the allele frequency test, and (ii) SNPTEST and QuickTest results differed from each other to an extent similar to their differences from the RegScan results.

**Table 1:** Pearson correlation coefficients between *P*-, β and SE values computed by SNPTEST (ST), QuickTest (QT) and RegScan (RS) based on 40 765 random markers

Parameter	RS versus QT	RS versus ST	QT versus ST
*P*-values	0.999998	0.999951	0.99995
β	1	0.999999	0.999999
SE	1	1	1

**Table 2:** Deviation (%) between *P*-, β and SE values computed by SNPTEST (ST), QuickTest (QT) and RegScan (RS) based on 40 765 random markers

Parameter	RS versus QT	RS versus ST	QT versus ST
Mean deviation of *P*-values (%)	0.119	0.114	0.006
*P*-values with >5% deviation (%)	0.000	0.010	0.010
*P*-values with >1% deviation (%)	2.956	2.951	0.010
Mean deviation of β (%)	0.018	0.017	0.036
β values with >5% deviation (%)	0.373	0.383	0.010
β values with >1% deviation (%)	1.820	1.828	0.010
Mean deviation of SE (%)	0.004	0.004	0.0002
SE values with >5% deviation (%)	0.000	0.007	0.007
SE values with >1% deviation (%)	0.000	0.010	0.010

The deviation (%) is calculated as the mean of the deviations of all markers (each calculated as the larger value divided by the smaller value times 100).

## EXAMPLES AND METHOD VALIDATION

We conducted proof-of-principle tests with 1000 Genomes-imputed 38.02 million markers [11], 873 individuals and 44 clinical traits of blood and all of their ratios to validate RegScan. The details are in Supplementary Data. In the first experiment, we tested whether RegScan was capable of detecting the associations well established by other tools in the other studies. For bilirubin, we identified the top three published markers with RegScan *P*-values <10^−^^50^ [1]. This shows that RegScan can function as a general GWAS tool. In the second test, we studied combinatorial traits involving plasma iron levels. For these combinatorial traits, we detected 20 markers that associated with trait ratios involving iron concentration at a genome-wide significance level. The candidates were ranked by RegScan based on a score that takes into account the *P*-values of the corresponding single traits. This score allows filtering of the hits based on statistical significance and is called here the Reliability Score, RS (Supplementary Data). The RS indicates how much stronger the association between the combinatorial trait and a given marker is compared with the corresponding single traits. The RS is calculated as RS = P_smaller_single_/P_combinatorial_, where P_smaller_single _is the *P*-value of association for the single trait that yielded the lower *P*-value of the two single traits; P_combinatorial_ is the *P*-value of association for the combinatorial trait. As an example, if P(A) = 10^−^^6^, P(B) = 10^−^^2^, P(A/B) = 10^−^^10^, then RS(A/B) = 10^−^^6^/10^−^^10 ^= 10^4^. We suggest that this simple score is effective in identifying the associations that are likely to be biologically more relevant. RegScan has functions to compute the RS and filter the results based on its value.

To validate the use of the RS in studying the combinatorial traits we conducted theoretical simulations that represent the theoretical ‘real-life situations’. We generated data for the scenario where the genetic marker affects a phenotype ratio as well as the scenario where the marker has linear effects on one or both phenotypes, but not on their ratio. The study indicated that the RS is able to identify the correct model in >95% of cases (details in Supplementary Data).

**Figure 3:**
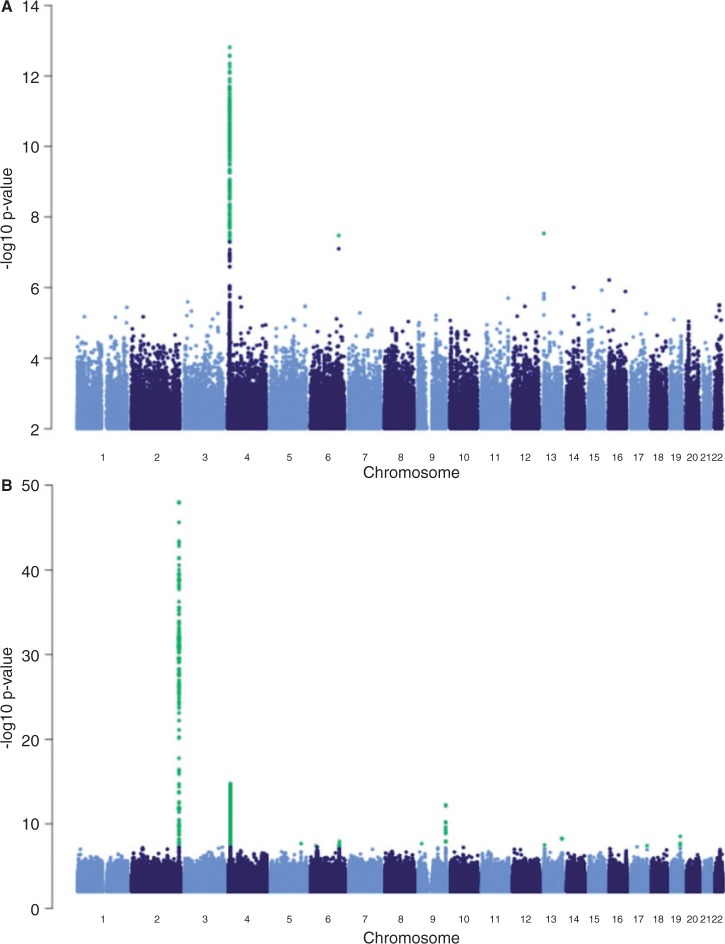
Manhattan plot showing the chromosome regions associated with blood plasma urate concentration (**A**), and with combinatorial traits involving urate concentration (**B**) as determined by RegScan.

A sample GWAS with the above data set was performed for blood serum urate concentration and all trait ratios that contained urate concentration. The relatively small number of individuals used in this example was sufficient to identify a known region in chromosome 4 [12]. However, using all trait ratios exposed at least four additional genomic regions (data not shown) that could be involved in urate metabolism in combination with some of the other 43 traits tested (Figure 3). This example highlights the power of using combinatorial traits by RegScan in detecting new candidate genomic regions for trait associations [5, 13].

## QUALITATIVE COMPARISON WITH THE OTHER TOOLS

The main advantages of RegScan include the following:

- Speed. Carrying out simple linear regression analysis maximally fast is the primary goal of RegScan, as it opens doors to studying large data sets.
- Unlike the reference tools tested, RegScan allows an automatic analysis of any number of traits at the same time. The user does not have to specify individual phenotypes to be analyzed. All phenotypes present in the input files are automatically analyzed against all markers present. This avoids the need to treat each trait separately and leads to major computational speedup.
- Easy creation of combinatorial traits. RegScan can conveniently convert phenotype files into combinatorial phenotype files.
- Two types of output files. In addition to the standard output listing statistical parameters for each marker, a summary information file can be created that finds the strongest-associating trait from among all traits tested for each marker. This is done based on (a) the statistical parameter selected by the user, or (b) the maximal effect size.
- Post-run data analysis for combinatorial traits. Several functions allow studying the association analysis results. We introduced a simple, yet useful, method (RS) for identifying associations with combinatorial traits based on the statistical parameters of the corresponding single traits.

The main disadvantage of RegScan is the absence of higher-level analytical functions in addition to the allele frequency association analysis. RegScan also relies on external R scripts for data adjustments.

## CONCLUSIONS

RegScan’s main focus is to find marker–trait associations in metabolomics in the context of combinatorial traits. Another predicted use is marker associations with gene expression. RegScan addresses the main obstacle in these studies—the heavy computational burden to find the main associations. RegScan is currently lacking several common analytical options. Our intent is to develop RegScan into a full-capability GWAS tool based on the user feedback.

## SUPPLEMENTARY DATA

Supplementary data are available online at http://bib.oxfordjournals.org/.

**Key Points**

- Depending on the data size, RegScan performs association analysis between markers and continuous traits ten to several hundred times faster than the other GWAS tools. Analyses that used to take weeks or months now take days.
- RegScan can automatically generate and analyze combinatorial traits; it can analyze any number of traits in one go.
- RegScan provides functions for filtering and additional analysis of the association analysis results; it introduces the concept of RS to study the combinatorial traits.
- RegScan is designed for metabolomics GWAS but is not limited to that.

Supplementary Data
