# Supplementary material for: RegScan: a GWAS tool for quick estimation of allele effects on continuous traits and their combinations
Source: Brief Bioinform. 2013 Sep 5;16(1):39–44. doi: 10.1093/bib/bbt066 (PMC4293375; doi:10.1093/bib/bbt066)
Supplement: Supplementary Data [file supp_16_1_39__index.html]

RegScan: a GWAS tool for quick estimation of allele effects on continuous traits and their combinations — Supplementary Data 

# RegScan: a GWAS tool for quick estimation of allele effects on continuous traits and their combinations

## Supplementary Data

files

**Files in this Data Supplement:**

- Supplementary Data - pdf file
- Supplementary Data - pdf file
