# Supplementary material for: RegScan: a GWAS tool for quick estimation of allele effects on continuous traits and their combinations
Source: Brief Bioinform. 2013 Sep 5;16(1):39–44. doi: 10.1093/bib/bbt066 (PMC4293375; doi:10.1093/bib/bbt066)
Supplement: Supplementary Data [file supp_bbt066_Appendix1.pdf]

Appendix 1

Supplementary Information

RegScan (Regression Scanner) 0.1

1. Reference tools

For reference we selected the commonly used tools that can perform linear regression analysis with allele frequency and continuous traits, and can output p-value, beta and se: SNPTEST (2.4.1) and QuickTest (0.97).

These are the command line arguments used:

SNPTEST

time snptest -data test.impute test.sample -method expected -pheno phenotype -frequentist 1 -o ST.out -use_raw_phenotypes

QuickTest

time quicktest –geno test.impute --snptest --pheno test.sample --method-mean --out QT.out --ignore-ties --no-progress --npheno phenotype

RegScan

time REGSCAN -M gwas -gfile test.impute -pfile test.regscan -out RS.out -statistic p -slope 0 -statlimit 1 -buffer 500

The initial speed tests indicated that RegScan was always the fastest followed by QuickTest. Therefore, QuickTest was used as reference in the computational speed tests.

There are also tools that perform linear regression analysis but do not output p-value (e.g. ProbABEL 0.3.0). Of all four tools tested, RegScan 0.1 always performed the fastest.

The computational speed tests were carried out on a single 64-bit 2.3 GHz AMD Opteron(TM) Processor on a computer cluster running Scienctific Linux 6.3 (Carbon) (Linux 2.6.32-279.5.1.e16.x86_64 x86_64). All tests were performed several times. Computational time was calculated as the sum of “sys” and “user” parameters returned by the “time” function (see below). Mean and standard error were calculated were applicable.

2. Speed as a function of the number of individuals tested

The analysis time of RegScan did not change relative to QuickTest when the number of individuals was varied. RegScan remained about 10 times faster with one trait. The computational time ranged from 0.08 – 0.34 msec/marker/trait for RegScan and 0.79 – 3.4 msec/marker/trait for QuickTest (figures are in the main article).

3. Speed as a function of the number of markers

We tested the computational speed of RegScan and QuickTest with 38.02 million markers, 1 trait, and 750 individuals. RegScan performed 10.6 times faster than QuickTest, therefore showing the same relative speed as with 1 million markers. The computational speed of RegScan was 0.073 msec/marker/trait under these conditions. [When the number of individuals was increased to 3315 (4.42 times higher) the analysis time increased 4.6 times – again showing linear relationship between the number of individuals and the analysis time.]

4. Speed per trait as a function of the number of traits

It is expected that the time spent on analyzing each trait is decreased with increasing the number of traits. This was tested with 5 million markers, 750 individuals, and variable number of traits. The results indicated that the analysis time decreased from 114 sec/trait with 112 traits to 56 sec/trait with 6212 traits, which corresponds to 0.011 msec/marker/trait.

5. Speed as a function of memory allocation

RegScan analysis can be further accelerated by allocating more memory for data reading.

The user can allocate 1 - n Mb of RAM for data reading. 1 Mb is sufficient with typical data sets to achieve most of the speed gain that RegScan features. We compared relative analysis speed with allocating 1 Mb vs. 1 Gb of RAM using the '-buffer' switch. The relative analytical speed 1 Gb / 1Mb was 13 % with 750 individuals, 38.02 million markers and 1 trait.

6. Eliminating less informative markers

RegScan analyis can be accelerated by removing the markers that have a low MAC (minor allele count) from the analysis. This is achieved by setting the MAC limit (-maclimit) higher. Fig. S2 shows relative processing time as a function of MAC threshold.

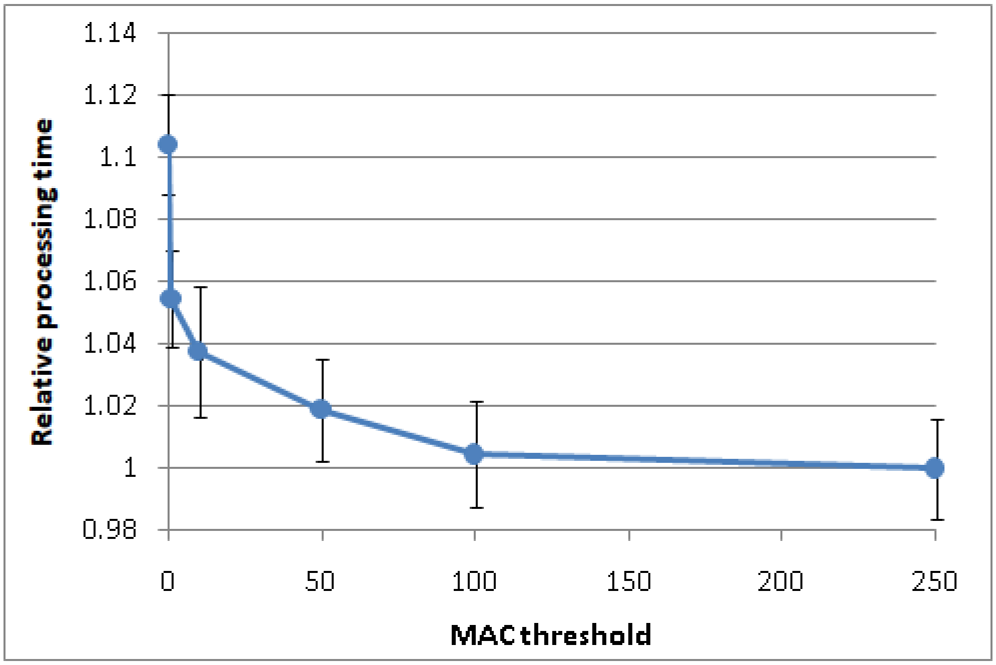

Fig. S2. Processing time (in relative units) and standard errors as a function of MAC threshold ('-maclimit'). Analysis carried out with 38.02 million markers, 873 individuals and 1 trait.

Analysis time can be shortened up to 11% by using the MAC filter ('-macfilter').

7. Speed of analyzing gzip files

Gzip files are typically analyzed about 6.5-6.8 times slower than the non-zipped files because unzipping takes time. In practice, if the input file is already gzip'ed, it is a better to analyze it directly than first unzip and then analyze because unzipping and file writing takes a significant amount of time.

8. Practical example to demonstrate RegScan analysis

We tested RegScan with a 1000 Genome imputed dataset of 38.02 million markers, 873 random individuals of European descent and 44 clinical traits to illustrate how RegScan functions. The traits were adjusted for gender and age and inverse-normally transformed. The ratio traits were created with RegsScan's “combitable” function. All marker-trait pairs (single or ratio values) with a p-value of association under 10^{-3} were written into the main output by the “gwas” function for further analysis performed by the other functions of RegScan. *This threshold was chosen arbitrarily for this example to ensure that the lowest p-values were not missed in decision-making (see below). All p-values could have been chosen instead but that would have yielded in very large file sizes. Since we are generally only interested in top hits the value of 10^{-3} works well.*

Below are two examples to serve only as proof of principle, they do not represent a scientific study.

1) Test if known trait-associated markers were identified by RegScan

We used bilirubin levels as the trait to compare the top RegScan-identified hits with the published bilirubin-associated markers to test if RegScan was able to detect any of the published markers. Our five top markers (rs111741722, rs887829, rs6742078, rs4148324, rs4148325) had a p-value under 10^{-50} and they included the topmost hit of each of the bilirubin-related association study published:

Datta S. et al, November 28, 2011, Ann Hum Genet

Chen G. et al, November 16, 2011, Eur J Hum Genet

Bielinski S.J. et al, June 06, 2011, Mayo Clin Proc

Sanna S. et al, May 06, 2009, Hum Mol Genet

Johnson A.D. et al, May 04, 2009, Hum Mol Genet

The results were also confirmed with QuickTest. The p-values, effect sizes and standard errors computed by QuickTest and RegScan agreed completely.

2) Identify markers for the trait ratios

We used blood plasma total iron concentration as the lead trait (one of the two traits in the trait ratio) in this example. This serves as a practical example to illustrate how RegScan can be used to identify significant markers.

We identified trait ratio candidates with RegScan's "combifilter" by setting the trait ratio p-value limit at $<5x10^{-8}$. For each marker the p-values of the single traits that corresponded to the trait ratio were compared and the smaller p-value was identified as the smaller single trait p-value (SSTP). Next a Reliability Score (RS) was computed for each pair of trait ratio and marker pair by dividing the SSTP by the p-value of the trait ratio (if the trait ratio p-value was $<5x10^{-8}$). All these steps are automatically performed by the "combifilter" function. The RS indicates how much lower the p-value of the trait ratio is compared to the "best" single trait. The higher RS values were considered more significant and all trait ratio and marker pairs were ranked according to the RS. The top hits based on the RS can be extracted as candidates. This method allows one to report a relatively short list of candidates for each trait. Here is an example for iron concentration:

MARKER	p-value of trait ratio	RS	Other trait
rs11250140	1.44E-08	69444	Gamma glutamyl transpeptidase
rs1600252	1.46E-08	68493	Gamma glutamyl transpeptidase
rs1600250	1.47E-08	68027	Gamma glutamyl transpeptidase
rs2736342	2.15E-08	46512	Gamma glutamyl transpeptidase
rs13272061	2.45E-08	40816	Gamma glutamyl transpeptidase
rs7822109	2.51E-08	39841	Gamma glutamyl transpeptidase
rs978804	2.63E-08	38023	Gamma glutamyl transpeptidase
rs4840567	2.63E-08	38023	Gamma glutamyl transpeptidase
rs978803	2.65E-08	37736	Gamma glutamyl transpeptidase
rs978802	2.73E-08	36630	Gamma glutamyl transpeptidase
rs7829381	3.02E-08	33113	Gamma glutamyl transpeptidase
rs1478890	3.41E-08	29326	Gamma glutamyl transpeptidase
rs17799486	4.62E-08	21645	Gamma glutamyl transpeptidase
chr18:71202594:D	2.86E-08	21224	Ferritin
rs11775150	4.73E-08	21142	Gamma glutamyl transpeptidase
rs11775149	4.74E-08	21097	Gamma glutamyl transpeptidase
rs7843880	3.32E-08	14789	Gamma glutamyl transpeptidase
chr2:234643095:I	9.71E-10	9928	Bilirubin
rs10929285	1.01E-13	6594	Bilirubin
rs74665357	3.40E-08	3706	Triglycerides

Note: In this example we used the p-value of $5x10^{-8}$ as the genome-wide significance level. Further filtering could be achieved by applying a Bonferroni-corrected p-value based on Principle Component Analysis.

3) Simulation study for the Reliability Score (RS)

To verify that the RS is able to select the correct trait transformation, we conducted a small simulation study, simulating three different scenarios with two trait variables, T_1 and T_2, and a genetic marker X (generated as an allele count variable with values 0, 1 or 2, with the probabilities corresponding to MAF=0.2 and Hardy-Weinberg equilibrium in the population):

1. Genetic marker X has a linear effect on the ratio T_1/T_2:

$T_1 = T_2\ (\beta_0 + \beta_1 X + \varepsilon)$,
with the variable T_2 having log-normal (μ=0, σ=1) distribution.

2. Genetic marker X has linear effects on both, T_1 and $T_{2,}$ whereas the effect on T_2 is stronger:

$T_1 = \beta_0 + \beta_1 X + \varepsilon_1$
$T_2 = \beta_0 + 2\beta_1 X + \varepsilon_2$

3. Genetic marker X has a linear effects on T_1 only (no effect on T_2):

$T_1 = \beta_0 + \beta_1 X + \varepsilon_1$
$T_2 = \beta_0 + \varepsilon_2$

Random error terms ε, ε_1, and ε_2 were generated as independent normal variates (μ=0,σ=3) and the parameters were set as β_0=10 and β_1=0.5.

For each of the scenarios, four regression models were fit: a linear model for T_1 on X, a linear model for T_2 on X, linear model for T_1/T_2 on X and a linear model for T_2/T_1 on X. For each model, a Wald p-value was obtained to test the trait-genotype association and the RS to compare different models were obtained. Table below summarizes the results of 10000 simulations as the percentage of cases when the ratio model had at least 10 times smaller p-value than the minimum p-value from models for T_1 and T_2 separately.

Scenario	T_1/T_2 preferred over T_1 or T_2	T_2/T_1 preferred over T_1 or T_2	T_1/T_2 preferred over T_2/T_1	T_2/T_1 preferred over T_1/T_2
1	99.5%	42.9%	97.6%	0.02%
2	0%	0%	-	-
3	0.03%	0.04%	-	-

In addition, different combinations of parameter values and error distributions were tested (results not shown), with the results remaining basically the same. Also, the scenarios were tested where the true model was multiplicative rather than linear. The RS statistic did prefer the ratio model over a linear single-trait model in about 7% of cases (although both models would be wrong), but convincingly preferred the correct log-linear model over all other alternatives.
