# Supplementary material for: RegScan: a GWAS tool for quick estimation of allele effects on continuous traits and their combinations
Source: Brief Bioinform. 2013 Sep 5;16(1):39–44. doi: 10.1093/bib/bbt066 (PMC4293375; doi:10.1093/bib/bbt066)
Supplement: Supplementary Data [file supp_bbt066_regscan_user_guide.pdf]

RegScan 0.1 Documentation

Website: www.biobank.ee/regscan.html
Contact: toomas.haller@ut.ee
Reference: *T. Haller, M. Kals, T. Esko, R. Mägi, K. Fischer. RegScan: a GWAS tool for quick estimation of allele effects on continuous traits and their combinations. Briefings in Bioinformatics.*

CONTENTS

I. INTRODUCTION – p. 2

II. HOW TO COMPILE THE PROGRAM – p. 3

III. GENERAL DESCRIPTION OF HOW RegScan WORKS – p. 3

IV. USER GUIDE – p. 4

1. Preparing the input files – p. 4
1.1 Preparing the genotype file – p. 4
1.2 Preparing the phenotype file – p. 5
1.2.1 Combinatorial traits – p. 5
1.2.2 Creating RegScan input – p.6
1.3 Data manipulations with R: transformations and adjustments – p. 7
2. Performing linear regression analysis – p. 8
2.1 Basic usage – p. 8
2.2 Choosing statistical parameters for filtering – p. 9
2.3 Output files – p. 10
2.4 Allocating memory – p. 11
2.5 Parallelization – p. 11
3. Analyzing results – p. 11
3.1 Counting and isolating markers of interest – p. 11
3.2 Counting and isolating traits of interest – p. 12
3.3 Evaluating combinatorial traits – p. 12
3.4 Additional filtering – p. 13
3.5 Identifying true positives – p. 13

APPENDIX – p. 14

4**. Detailed overview of functions – p. 14**
4.1 “gwas” function – p. 14
4.2 “singlemath” function – p. 14
4.3 “extract” function – p. 15
4.4 “transpose” function – p. 15
4.5 “combitable” function – p. 16
4.6 “fragmentrows” function – p. 17
4.7 “changedelim” function – p. 17
4.8 “remseqdup” function – p. 18
4.9 “multicount” function – p. 18
5.0 “combifilter” function – p. 19
5.1 “multifilter” function – p. 20
5.2 “extractid” function – p. 22

V. WORKED EXAMPLE – p. 23

I. INTRODUCTION

RegScan is an open source command line tool (available under GNU GPL) for performing fast analysis of genotype and phenotype data. It uses linear regression to estimate marker effects on continuous traits. Filtering of results is done using a) the slope (effect size), b) standard error of slope, c) R^2, t-value, or p-value, and d) MAC (minor allele count).

RegScan's main goal is to achieve maximal computational speed in order to be applicable for testing of very large datasets. RegScan does not pretend to be an all-inclusive GWAS solution but rather a quick screening tool. Currently it is about an order of magnitude faster than the leading GWAS methods (SNPTest, QuickTest, ProbABLE) but the speed gain varies with the data size and type. Compared to QuickTest it performs about 10 times faster with one trait. The relative speed gain per trait is significantly higher (several hundred times) than that when large numbers of traits are analyzed together or when various restrictive filters are set.

The GWAS data sets can be large due to a large number of markers or individuals, or a multitude of traits tested. In an attempt to link the GWAS data to the biological mechanisms an increasing amount of effort goes into studying the marker effects on combinatorial traits such as trait ratios. This results in a very large number of tests to be performed, which is often beyond the capabilities of the available computational resources. RegScan is designed to deal with the combinations of traits (such as ratios). It can both generate the combinatorial traits and rapidly screen them to isolate the potentially interesting ones for further research.

RegScan achieves its speed by efficient implementation and performing only a critical number of statistical tests. RegScan comes with several supporting functions required for data file preparation and conversion as well as post-runtime analysis. It is an open source project; the code can be compiled for all major computational platforms, and both the 32- and 64-bit hardware architectures.

II. HOW TO COMPILE THE PROGRAM

You can download the program and instructions form the website (www.biobank.ee/regscan.html). The statically compiled versions are ready to run, dynamically compiled versions require that Qt libraries are installed on your system. Qt libraries are free and can be downloaded and installed from http://qt-project.org/downloads.

In order to compile RegScan from the source code, place the source code in the folder named "REGSCAN" on the Qt path and execute the following lines:

qmake -project
qmake
make

Note1: If you are compiling for Windows, open your *.pro file after typing "qmake -project" and add this line to the *.pro file:
CONFIG+=console
INCLUDEPATH += C:\Qt\4.4.3\src\3rdparty\zlib (where C:\Qt\4.4.3\ should be replaced with the correct path)

Your *.exe file will appear in the "release" subfolder.

Note2: If you are compiling for Mac, you may need to have Xcode installed (https://developer.apple.com/xcode/) if your computer doesn't already have it. Before you install, look under "/Applications" to see if "/Applications/XCode" exists.

If you installed Qt on a Mac using binaries, the program should be located here:
"~/QtSDK/Desktop/<qt.version>/<compilerversion>/bin", if you built it from source code, it should be here:
"/usr/local/Trolltech/<qt.version>/bin".

Note3: If you are compiling for Mac you need to execute the following lines in order to compile using g++:
qmake -project
qmake -spec macx-g++
make

After you have created your Makefile (before typing "make") you may need to add "-lz" after "LFLAGS=" in your Makefile.

Note4: If you are compiling statically, open your *.pro file after typing "qmake -project" and add this line to the *.pro file:
CONFIG+=staticlib

Static compilation is an option only if Qt itself was built statically. Static compilation is not generally recommended.

If you have any problems compiling or running RegScan, please do not hesitate to contact the author at tom@toomashaller.com for help.

III. GENERAL DESCRIPTION OF HOW RegScan WORKS

To run RegScan first open your terminal and navigate into the folder where the RegScan executable resides. For the ease of use you can put your input files in the same folder with RegScan but this is not a requirement.

Note: *Depending on how the executable was created, you may need to change the file permissions (rwx settings) to allow execution. With unix-like architectures all permissions are granted by typing "chmod ugo+rwx REGSCAN".*

RegScan accepts valid switches (flags) in any order. However, each switch needs to be followed by exactly one argument (which may be a composite argument but without spaces, see below), separated by a space. When a switch is not used, its default value is used. Some switches are optional but a few are mandatory. Each function has its own mandatory switches (see below) but the '-M' switch is always required, it defines the function (method) to be used.

Examples for running RegScan on various systems:

Linux: **./REGSCAN -M** *myfunction*
Windows: **REGSCAN.exe -M** *myfunction*
Mac: **open REGSCAN.app -M** *myfunction*
or
open -a REGSCAN -M *myfunction*

Note: *The RegScan functions are optimized for handling very large data files (they have a minimal memory footprint and are fully scalable).*

IV. USER GUIDE

The text below is for sequential reading as it introduces the switches one by one. It will walk you through all the functions of RegScan. Please do not skip this section and read through the examples as some of the information is provided only through examples. The command line examples are given for Linux. Following the user guide, you'll find worked examples as well as the detailed function descriptions.

1. Preparing the input files

RegScan uses two input files: **a)** genotype data in the Oxford GEN format (.gen or .impute); same as SNPTEST, **b)** phenotype file (derived from the Oxford SAMPLE format). If you have a .gen/.sample pair, you are ready to go. If you have a .ped/.map files or .bed/.bin/.fam files, you can convert them using GTOOL and PLINK.

Note: *GTOOL can be downloaded here:* http://www.well.ox.ac.uk/~cfreeman/software/gwas/gtool.html
Note: *PLINK can be downloaded here:* *http://pngu.mgh.harvard.edu/~purcell/plink/download.shtml*

1.1 Preparing the genotype file

RegScan uses .gen files as the input format for genotype data. The same format is used by SNPTEST and QuickTest and is described here: http://www.stats.ox.ac.uk/~marchini/software/gwas/file_format.html. You don't need to modify this format for RegScan in any way.

RegScan can read and analyze normal .gen files or their gzip-compressed versions. It automatically recognizes if the file is gzip'ed or not by searching for ".gz" at the end of the file name.

1.2. Preparing the phenotype file

The phenotype file should contain one trait per row, all individual values next to one another, separated by space. Each row starts with the trait ID (no spaces allowed in the name), followed by the individual phenotype values in the same order as in the genotype (.gen) file:

Example:

Height 1.73 1.64 1.91 …
Weight 69.7 51.2 88.4 …
… … … … ...

Note that this (.regscan) format is different from the .sample format. Below is a description for converting the sample format into the .regscan format (please also refer to the worked example at the end of this document).

To isolate the required rows and columns from a tabular .sample file use the “extract” function. Here's an example for a typical .sample file (for 100 individuals) with an “oops” line to be removed (2nd row) and phenotypes located in columns 4 through 9:

./REGSCAN -M *extract* **-rows** *1,3-102* **-columns** *4-9* **-file** *phenos.sample* **-out** *phenos.txt*

Note: *Row or column ranges are denoted with a “-” and are separated by commas (no spaces). Space is the default delimiter. If your delimiter is not space but something else, such as TAB, you need to tell that to RegScan by typing '-delim tab'. See Appendix for full functionality of the “extract” function. If you use a unix-like system, this and other similar steps can also be performed with the unix commands.*

The resulting file (phenos.txt) has a header with the trait identifiers followed by rows containing trait values for all individuals (one row for each individual). At this point you have two options:
a) If you want to analyze combinatorial traits you need to create these traits by applying the “combitable” function (see 1.2.1),
b) If you want to analyze the simple traits you already have, you can skip 1.2.1 and proceed directly to creating RegScan trait file by using the “transpose” function (see 1.2.2).

1.2.1 Combinatorial traits

If you are using RegScan to analyze combinatorial traits, RegScan can generate the combinations for you. The “combitable” function generates all combinations of traits (ratios, products, sums, or differences). Since the full combinatorial matrix is diagonally symmetrical from the viewpoint of regression analysis, only one half of the matrix (upper right) is computed and the diagonal is excluded. For example, 4 phenotypes will give you 6 unique combinations (while the full size matrix would be 16 entries).

Note: *The input format for the “combitable” function is such that the traits are organized in columns (top to bottom, similar to the .sample file). The file can have an optional header row showing the phenotype names, but the oops line (the second line) of the .sample file should be removed.*

To obtain trait ratios, use the following command line:

./REGSCAN -M combitable **-file** phenos.txt **-out** pheno_combi.txt **-function** divide **-header** yes

In this example the trait ratios are performed by combinatorially dividing the values ("phenos.txt" was prepared in section 1.2).

Note: *For the combinatorial traits to be meaningful and comparable, it is best to have the values all with the same sign and preferably of similar magnitude (see also below). Applying the ln or log transformation is often used to obtain distributions more similar to normal distribution. RegScan is capable of this and several other such transformations (please see the "singlemath" function in the Appendix).*

The ratios that cannot be created (for example division by zero errors) are filled in as missing values. The default missing value identifier is "NA" but this can be changed by adding '-missing PARAMTER' switch where PARAMETER is the missing value:

./REGSCAN -M combitable **-file** phenos.txt **-out** pheno_combi.txt **-function** divide **-missing** novalue **-header** yes

In this case the new missing value is "novalue".

Note: *The '-header' tag indicates that a header is used. When header is used the new phenotype names are logically derived from the original names. When a header is not present, new names are logically created based on their index number. See Appendix for full functionality of the "combitable" function.*

After you have generated the combinatorial traits you can proceed to transposing the file (see 1.2.2) to generate RegScan input. This work flow is also illustrated with the aid of a practical example at the end of this user guide.

1.2.2 Creating RegScan input

RegScan requires that phenotype values are located in the rows not columns. We therefore need to transpose (flip to the side) the file just created in the previous examples (1.2, 1.2.1). This is done with the "transpose" function:

./REGSCAN -M transpose **-file** phenos.txt **-out** phenos_transposed.txt
./REGSCAN -M transpose **-file** pheno_combi.txt **-out** phenos_transposed.txt

Note: *See Appendix for full functionality of the "transpose" function. This function can perform sequential transposition which makes it capable of transposing files of any size (including very large files that do not fit in the RAM). It can accept other delimiters than space (defined using the -delim switch).*

Finally, if your phenotype file is not space delimited it must be converted to that. To change the file delimiter you can us the "changedelim" function:

./REGSCAN -M changedelim **-file** phenos_transposed.txt **-out** phenos_ready.txt **-delim1** comma **-delim2** space

Note: *See Appendix for full functionality of the "changedelim" function.*

1.3 Data manipulations with R: transformations and adjustments

In order to be able to apply standard filters (threshold limits) to selecting relevant hits with RegScan the trait data need to be prepared in a certain way. When comparing results it is important that the trait values are standardized; this is especially important with trait ratios. We recommended to use inverse normal transformation.

RegScan cannot directly adjust trait values with regard to covariates or perform inverse normal transformation. This needs to be done before subjecting the data set to RegScan's linear regression analysis. Using R is a convenient way to achieve that. Below is a standard R script for adjusting and transforming raw data set for RegScan's linear regression analysis:

```
#column number where traits start (change)
startpos=10

#column number where traits end (change)
endpos=100

#input file name (change)
#input file has a header and is space-delimited
data<-read.table("input.txt",sep=" ",header=T,strings=F)

n <- names(data)

for (i in startpos:endpos) {

  #list of covariates to be adjusted for (change)
  model <- lm(data[,i] ~ cov1 + cov2 + cov3, data=data)

  res <- resid(model)
  QnormRes <- qnorm ( (rank(res, na.last="keep")-0.5) / sum(!is.na(res)) )
  data<-cbind(data,QnormRes)

}

names(data)[(endpos+1):(endpos+1+endpos-startpos)] <- n[startpos:endpos]

#output file name (change)
write.table(data,file="output.txt",sep=" ",row.names=F,quote=F)
```

This script appends the adjusted/transformed data (columns) to the original data. The new data are in columns 101-190 and they need to be extracted with the RegScan function "extract" (if the entire table does not need to be used) and transposed with "transpose". Please see section 1.2 (above) for more details.

Note: *You need to have the covariates that you adjust for in the same file with your data (located in columns).*

2. Performing linear regression analysis

2.1 Basic usage

Linear regression analysis is evoked with “-M gwas”. Here's the simplest use with the default settings:

./REGSCAN -M gwas **-pfile** phenofile.txt **-gfile** genofile.txt **-out** results.txt

The phenotype and genotype files are defined with the '-pfile' and '-gfile' switches, respectively**.** Both files need to be space delimited. If they are not, you can change file delimiter with the “changedelim” function (see above or below).

The '-out' switch defines the output file name that contains the list of all markers that pass the threshold filters (see below). The '-out' switch is optional, if it is not used the name is derived by adding “.out” to the phenotype file name.

It is required that the genotype file does not contain missing data. However, your phenotype data may contain missing values. If the missing values are marked with “NA” (capital letters) the program knows to exclude them from the analysis. If another identifier is used, it needs to be declared using the '-missing' switch.

Example:
./REGSCAN -M gwas **-pfile** phenofile.txt **-gfile** genofile.txt **-out** results.txt **-missing** absent

Here the word “absent” is treated as an indicator of missing data.

The previous example generates output by the marker. If you are working with multiple traits and you would like to see the best-hit marker for each trait, turn on the summary option by setting the '-summary' switch to “yes”.

Example:
./REGSCAN -M gwas **-pfile** phenofile.txt **-gfile** genofile.txt **-out** results.txt **-summary** yes

Now an additional output file is created. Its name is derived from the output file name by appending (.stats) to it. This file lists for every trait (or combinatorial trait) what the best marker was according to a) the absolute slope, b) the selected statistic (see below). Creating this file will slow down the execution somewhat but the resulting file can be useful for quickly finding the best marker for each trait tested.

2.2 Choosing statistical parameters for filtering

For each linear regression event RegScan always reports the slope (effect size) and R^2 value. It is also possible to display the standard error of the slope (SE), t-value and p-value. The default behavior of RegScan is to report all of the above. For the sake of computational speed gain, some of the parameters can be turned off:

./REGSCAN -M gwas **-pfile** phenofile.txt **-gfile** genofile.txt **-out** results.txt **-statitstic** r2
./REGSCAN -M gwas **-pfile** phenofile.txt **-gfile** genofile.txt **-out** results.txt **-statitstic** t
./REGSCAN -M gwas **-pfile** phenofile.txt **-gfile** genofile.txt **-out** results.txt **-statitstic** p

The first option reports only R^2, the second option reports R^2 and t-value, the third option reports R^2, t-value, and p-value (and is the default if the '-statistic' switch is not used).

Note: *The lower limit for p-value is 1e-50. All p-values below this number are reported as 1e-50.*

Separating interesting hits from less interesting results is the reason for running linear regression. RegScan offers four parameters that can be used to decide what hits are written into output files and what not. Filtering is based on: **a)** absolute slope (effect size), **b)** statistics that can be used to evaluate the goodness of fit between the data points and linear model or probability (R^2 statistic, t-value, p-value), **c)** the standard error of slope, **d)** MAC (minor allele count).

* The '**-slope**' switch retains all hits with the absolute slope values greater than the parameter value.
* The '**-selim**' switch eliminates all entries that have the standard error of slope larger than the value specified.
* The '**-statlimit**' switch retains all hits that (depending of which statistic is used) have :

$R^2 \geq$ *reference value*
t-value $\geq$ *reference value*
p-value $\leq$ *reference value*

* The '**-maclimit**' switch forces RegScan to only consider the markers that meet or exceed a minimal minor allele count threshold value.

Examples:

./REGSCAN -M gwas **-pfile** phenofile.txt **-gfile** genofile.txt -**statistic** r2 **-statlimit** 0.9 **-slope** 0.1

Here all candidates are selected that have a slope value larger than 0.1 or smaller than -0.1 AND R^2 value over 0.9

./REGSCAN -M gwas **-pfile** phenofile.txt **-gfile** genofile.txt -**statistic** p **-statlimit** 1e-3 **-slope** 0.1

Here all candidates are selected that have a slope value larger than 0.1 or smaller than -0.1 AND p-value under 0.001

./REGSCAN -M gwas **-pfile** phenofile.txt **-gfile** genofile.txt -**statistic** t **-statlimit** 0.1 **-selimit** 1.5 **-slope** 0.1

Here all candidates are selected that have a slope value larger than 0.1 or smaller than -0.1 AND t-value over 0.1 AND standard error of y value under 1.5

./REGSCAN -M gwas **-pfile** phenofile.txt **-gfile** genofile.txt -**statistic** t **-statlimit** 0.1 **-selimit** 1.5 **-slope** 0.1 **-maclimit** 3

The same as above but only the markers that have a MAC equal to or over 3 are considered.

2.3 Output files

If the '-summary' switch is not turned on RegScan produces one output file with one marker per row followed by the phenotype name and the statistical values:

Col1: marker name
Col2: marker position
Col3: phenotype (trait) name
Col4: number of phenotypes
Col5: number of individuals with the AA genotype
Col6: number of individuals with the AB genotype
Col7: number of individuals with the BB genotype
Col8: MAC (minor allele count)
Col9: slope (effect size)
Col10: standard error of slope (SEslope); not present if '-statistic r2' selected
Col11: R^2 value
Col12: t-value; not present if '-statistic r2' is used
Col13: p-value; not present if '-statistic r2' or '-statistic t' is used

If the '-summary yes' option is used, an additional summary file is produced that presents the best trait for each marker. This option should only be used if multiple traits, such as trait ratios, are tested:

Col1: marker name
Col2: marker rank number in the genotype file
Col3: number of trait hits for the current marker
Col4: the trait that returned the highest effect size for the current marker
Col5: absolute slope value for the trait in column 4
Col6: statistic (R^2, t-value or p-value) for the trait in column 4 (statistic is chosen with the '-statistic' switch)
Col7: the trait that returned the best statistic value for the current marker
Col8: absolute slope value for the trait in column 7
Col9: statistic (R^2, t-value or p-value) for the trait in column 7 (statistic is chosen with the '-statistic' switch)

2.4 Allocating memory

A significant speed gain of RegScan comes from the fact that read/write events are optimized and data are read in maximally efficiently. The more memory can be allocated for these processes the faster RegScan is. The user can change how much memory in megabytes (Mb) is allocated for data reading. This is done using the '-buffer' switch.

```
./REGSCAN -M gwas -pfile phenofile.txt -gfile genofile.txt -buffer 200
```

Here 200 Mb is allocated for reading the data from the genotype file (data are read into the buffer in 200 Mb chunks). The more memory you can allocate the faster the analysis. The default value is 100 Mb.

Note: *This switch controls only the memory allocated for data reading. RegScan uses memory for other functions as well; additionally your computer needs memory for other functions. As the rule of thumb, do not allocate more than 50% of your total memory to the read function using '-buffer'.*

2.5 Parallelization

RegScan does not currently have automatic parallelization. If you want to run the analysis in parallel, it is recommended to fragment the genotype file with RegScan function "fragmentrows" (see below) and analyze the fragments in parallel by manually setting up the parallel analyses.

3. Analyzing results

The main output file of RegScan "gwas" function contains information that can be used in additional analyses. Below are some examples of how RegScan could be used a) to find out what markers were "pulled out" of the initial marker list (.gen/.impute) by the traits used in the analysis (3.1), b) how the frequency of encountering a trait name among the trait hits could be used to add weight to its potential importance among the top hits (3.2), c) how the p-values of combinatorial traits could be used in combination with the p-values of the corresponding single traits to estimate the reliability of hits (3.3), d) how the data can be additionally filtered (3.4).

3.1 Counting and isolating markers of interest

This function may be useful when RegScan is used to narrow down the list of potentially interesting markers for a next round of analysis with a another tool. For example when thousands of trait ratios were used and we want to know what marker names were found among all hits. The marker list (produced by "gwas" function) could be isolated and duplicates removed to obtain a list of potentially interesting markers and therefore compress the initial dataset. RegScan function "remseqdup" can provide a compressed list of all markers and also report how many times each marker was encountered:

./REGSCAN -M remseqdup -file gwasoutput.txt **-out** results.txt **-delim** tab **-header** yes

Note: *Please see Appendix for full functionality of the "remseqdup" function.*

3.2 Counting and isolating traits of interest

The “multicount” function counts the occurrences of ID's in a specific column of a file based on a reference list given in another file. In essence, it attaches a number to each entry in the reference list stating how many times this entry was encountered in the main file. It can also break the entries into fragments based on the presence of an additional delimiter when '-iddlim' is selected. For example “waist/hip” (listed in “references.txt”) is normally considered to be one ID:

./REGSCAN -M multicount -file myfile.txt **-out** results.txt **-list** references.txt **-delim** tab **-column** 2 **-header** yes

However, when '-iddelim /' is set it is considered as two separate ID's: “waist” and “hip”:

./REGSCAN -M multicount -file myfile.txt **-out** results.txt **-list** references.txt **-delim** tab **-iddelim** / **-column** 2 **-header** yes

This function requires a reference file that lists all ID names (one per row) that you want to count. If you want to study all traits then this list is the same as the first column of your .regscan file (see above), This column can be extracted with the “extract” function.

Note: *Please see Appendix for full functionality of the “multicount” function.*

3.3 Evaluating combinatorial traits

When working with combinatorial traits such as trait ratios the ratios can be considered interesting if they have a selection parameter (such as the p-value) “better“ than that of the nominator trait and/or the denominator trait alone. RegScan can help to find these combinations by performing “combifilter” analysis. The “combifilter” function allows to compare the analysis results of single traits with those of the combinatorial traits (involving the single traits) based on some chosen statistic (such as p-value). If a combinatorial trait yields a lower p-value in linear regression analysis than one or two of the single traits alone, it is considered interesting and is reported in the output alongside with a number showing whether the combinatorial trait was “better” than one or both of its constituent traits. The “combifilter” function allows to define “better”:

Example:
./REGSCAN -M combifilter **-file** myfile.txt **-out** results.txt **-delim** tab **-iddelim / -dir** smaller **-threshold** 5e-8 **-factor** 10 **-missing** NA

Here only the ratio traits that had a p-value smaller than 5e-8 AND the p-value was 10 times smaller than that of one or both of the single traits are reported (included in the output file).

Note: *Please see Appendix for full functionality of the “combifilter” function. More parameters are needed when working with files other than the main output of “gwas”.*

Please note that for this analysis to work you cannot use narrow filtering options when you perform “gwas”. Also be advised that the input files used for this analysis may be very large. The output file generated serves as a good starting point to further narrow down the list of potentially interesting

combinations (see below).

3.4 Additional filtering

RegScan can filter results during runtime (“gwas” function) or after performing “gwas” (“multifilter” function). Filtering during runtime has the advantage of accelerating the analysis and reducing the file size. Post-runtime filtering allows to narrow down the options even further. The “multifilter” function can do the same filtering as “gwas” but is much more powerful. It allows setting unlimited number of filters. “Multifilter” can be efficiently used to further filter the files created in section 3.3.

Example:
./REGSCAN -M multifilter -file mydata.txt **-out** results.txt **-delim** tab **-missing** NA **-ignore-missing** no **-logic** and **-dir** include **-header** yes **-filter** abs:Slope_l_0.01,P_se_5e-8,P_ne_NA

In this elaborate example all hits are included in the output file that have their absolute slope value larger than 0.01 AND p-value smaller than or equal to 5e-8 AND the p-value is not missing (“NA”).

Note: *Please see Appendix for full functionality of the “multifilter” function. Appendix also describes how to construct the logical filters. Individual filters are separated by commas. Each filter is constructed by using parameters separated by “_”. For example “P_ne_NA” means “if P does not equal to NA”; “abs:Slope_l_0.001” means “if absolute value of Slope is larger than 0.01”.*

When you work with combinatorial traits all results are placed in one file. You may want to isolate only the hits involving a certain keyword or certain ID in the trait name, for example.

./REGSCAN -M extractid -file mydata.txt **-out** results.txt **-column** 2 **-contains** biotin

In this example only the rows that contain “biotin” in column 2 are isolated and put in the output file.

Note: *Please see Appendix for full functionality of the “extractid” function.*

3.5 Identifying true positives

Depending on the data set and the settings RegScan's “gwas” function may return a large number of potential hits. Identifying true positives is always a challenge and is specific to each case. Some additional methods of identifying true positives (not elaborated here further) include:

a) Use the Bonferroni correction to find the statically justified p-value threshold
b) Apply a false discovery rate (FDR) technique to filter out the most reliable hits
c) Use permutation testing to establish statistical significance levels for filtering
d) Use LD information or biological information

APPENDIX

4. Detailed overview of functions

4.1 “gwas” function

This function performs linear regression with genotype and trait data. It is the core function of RegScan. Hit filtering is performed according to the parameters defined by the '-slope', '-statlimit' '-selimit', and '-maclimit' switches.

Prototype: **./REGSCAN -M gwas -gfile -pfile -missing -slope -statistic -statlimit -selimit -maclimit -out -summary -buffer**

-gfile = genotype file in the .gen/.sample format. REQUIRED.
-pfile = phenotype file in the .regscan format (see above). REQUIRED.
-missing = what is used to indicate the missing values. Options: any symbol or word without spaces. Default = "NA".
-slope = minimal absolute slope value required for a marker to be reported in the output. Default = 0.
-statistic = the primary statistic used for screening. Options: r2 (for R^2),t (for t-value), p (for p-value). Default = p.
-statlimit = minimal (R^2, t-value) or maximal (p-value) value for the statistic to be reported in the output. Defult R^2 = 0, default t-value = 0, default p-value = 1.
-selimit = maximal allowed standard error of y value for the marker to be reported in the output. Default = 1e12.
-maclimit = minimal allowed minor allele count. Default=0.
-out = output file name. Default = genotype file name + ".out".
-summary = produces an additional output file where best traits are attributed to each marker (see above). Options: “yes”, “no”. Default = “no”.
-buffer = memory (in Mb) allocated for reading the genotype data. Default = 100 Mb. The larger this value the faster the function performs. Note: It is important to not allocate more resources than your computer has.

Example:

./REGSCAN -M gwas -gfile genotypes.gen **-pfile** phenotypes.regscan **-missing** na **-statistic** p **-slope** 0.05 **-statlimit** 5e-8 **-selimit** 1.2 **-maclimit** 2 **-out** results.txt **-summary** yes **-buffer** 500

4.2 “singlemath” function

This function performs exp, ln, log, sqrt, square, inv, rev on any or all columns of your table.

Prototype: **./REGSCAN -M singlemath -file -function -columns -missing -header -delim -out**

-file = input file name. REQUIRED.
-function = mathematical function. REQUIRED. Options: "exp", "ln", "log", "sqrt", "square", "inv" (inverse), "rev" (reverse sign).

-columns = what columns to use. REQUIRED. Notes: columns should be separated by commas and ranges by dashes (see the example below); there should be no spaces. If you want all columns, use wide enough range (which is allowed to exceed the size of the file).
-missing = what is used to indicate the missing values. Options: any symbol or word without spaces. Default = "NA".
-header = file header. Options: "yes", "no". Default = "no".
-delim = file delimiter. Options: a) "tab", "space", "comma", "semicolon", "colon", b) any symbol or word without spaces. Default = "space".
-out = output file name. Default = input file name + ".out".

Example:
./REGSCAN -M singlemath -file myfile.txt **-function** ln **-columns** 2,4-6 **-missing** na **-header** yes **-delim** comma **-out** results.txt

Natural logarithm is taken from values located in columns 2,4,5,6 but not in the header (first) row.

4.3 “extract” function

This function extracts individual columns or rows, or their ranges (both continuous and non-continuous) from a tabular text file. If the user-defined range is longer than the file, the limit is automatically set to file size.

Prototype: **./REGSCAN -M extract -file -columns -rows -delim -out**

-file = input file name. REQUIRED.
-columns = columns to be extracted. REQUIRED. Note: columns should be separated by commas and ranges by dashes (see the example below); there should be no spaces.
-rows = rows to be extracted. REQUIRED. Note: rows should be separated by commas and ranges by dashes (see the example below); there should be no spaces. Header (if your file has one) is considered a normal row (row=1).
-delim = delimiter. Options: a) "tab", "space", "semicolon", "colon", "comma", b) any symbol or word without spaces. Default = "space".
-out = output file name. Default = input file name + ".out".

Example:
./REGSCAN -M extract -file myfile.txt **-columns** 1,7-9 **-rows** 4,7 **-delim** space **-out** results.txt

Columns 1,7,8,9 and rows 4,7 are extracted from myfile.txt. The rows and columns retain their relative order regardless of how they are defined. e.g. 1,2,3 gives the same order as 3,1,2.

4.4 “transpose” function

This function transposes a table (swaps columns with rows). The process can be carried out any number of columns at a time. For small tables transposing all columns in one go gives maximal speed. However, very large tables may be too large (file sizes near the computer RAM limit) to fit in the

computer memory; these tables need to be transposed a smaller number of columns at a time. This function allows to select the maximum number of columns stored in the memory during transposition. This approach allows handling very large tables (data sets). Note: the capability to handle very large files comes at the cost of reduced speed.

Prototype: **./REGSCAN -M transpose -file -columns -col-chunk -delim -out**

-file = input file name. REQUIRED.
-columns = number of columns in the table. Options: any positive integer, 0 (means that max column number is found automatically), -1 (means that the column number is calculated based on the first row). Default = 0.
-col-chunk = number of columns transposed in one iteration. Options: any integer. Default = 100. Note: Use larger values for smaller data sets and smaller numbers for very large data sets.
-delim = file delimiter. Options: a) "tab", "space", "comma", "semicolon", "colon", b) any symbol or word without spaces. Default = "space".
-out = output file name. Default = input file name + ".out".

Example:
./REGSCAN -M transpose -file mytable.txt **-columns** 0 **-col-chunk** 1000 **-delim** space **-out** results.txt

The file is transposed 1000 columns at a time, maximal column number is used as the width of the column (-columns 0).

4.5 "combitable" function

This function adds new columns to a table by using the existing columns. The new columns are created by dividing, multiplying, adding, or subtracting columns from one another. The combinations are created in a non-overlapping manner (i.e. only a half of the pairwise combinations matrix are created, excluding the diagonal). If the table has a horizontal header, the header is used to logically generate names for the new columns. If the header does not exist, the names are automatically generated and they consist of integers and their combinations. The data need to be organized in columns similar to the .sample file format.

Prototype: **./REGSCAN -M combitable -file -columns -function -header -delim -missing -out**

-file = input file name. REQUIRED.
-columns = columns used for creating new columns. REQUIRED. Options: any valid column numbers or ranges without spaces (Ex: 1,3-5,7 means that columns 1,3,4,5,7 will be used in a combinatorial manner).
-function = formula for creating new columns. Options: "divide", "multiply", "add", "subtract". Default = "divide".
-header = input file header. Options: "yes", "no". Default = "no".
-delim = file delimiter. Options: a) "tab", "space", "comma", "semicolon", "colon", b) any symbol or word without spaces. Default = "space".
-missing = what is used to indicate the missing values. Options: any symbol or word without spaces. Default = "NA".

-out = output file name. Default = input file name + ".out".

Example:
./REGSCAN -M combitable -file mytable.txt **-columns** 1,5-8 **-function** divide **-header** yes **-delim** tab **-missing** novalue **-out** results.txt

Columns 1,5,6,7,8 are used in a combinatorial way to generate new columns containing ratios. The header for the new columns is generated by using the original header names. The missing value indicator is "novalue", the delimiter is tab.

4.6 "fragmentrows" function

This function allows to automatically divide a file into smaller parts by selecting the numbers of rows for the chunks. The last chunk may be smaller than the selected chunk size if the original file size is not a multiplicate of the the selected size. The new files are automatically given sequential index-containing names. This function can be used to quickly fragment genotype file for simple parallelization of the "gwas" function.

Prototype: **./REGSCAN -M fragmentrows -file -fragmentsize -style -out**

-file = input file name. REQUIRED.
-fragmentsize = the number of rows per each new file. Options: any positive integer. Default = 1.
-style = how the new files are named. Options: "prepend", "append". Default = "prepend". Note = This is a way to determine if file indexes are at the beginning or end of the file name.
-out = output file name. Default = input file name + ".out".

Example:
./REGSCAN -M fragmentrows -file myfile.txt **-fragmentsize** 10000 **-style** append **-out** results.txt

Myfile.txt is fragmented into chunks containing 10000 rows. The numerical index in the file name indicating the chunk order is at the end of the file name.

4.7 "changedelim" function

This function changes the file delimiter.

Prototype: **./REGSCAN -M changedelim -file -delim1 -delim2 -out**

-file = file name. REQUIRED.
-delim1 = original file delimiter. REQUIRED. Options: a) "tab", "space", "comma", "semicolon", "colon", b) any symbol or word without spaces. Default = "tab".
-delim2 = original file delimiter. REQUIRED. Options: a) "tab", "space", "comma", "semicolon", "colon", "none", b) any symbol or word without spaces. Default = "tab". Notes: "none" will remove the previous delimiter.
-out = output file name. Default = input file name + ".out".

Example:
./REGSCAN -M changedelim -file myfile.txt **-delim1** tab **-delim2** space **-out** results.txt

4.8 “remseqdup” function

This function removes duplicates from an ordered list. The list needs to be ordered so that the identical ID's are below one another. This requirement is fulfilled when using the marker names from the RegScan funtion “gwas” main output file. Tis function also counts how many times each ID was encountered.

Prototype: **./REGSCAN -M remseqdup -file -out -delim -column -header**

-file = file name. REQUIRED.
-out = output file name. Default = input file name + ".out".
-delim = file delimiter. Options: a) "tab", "space", "comma", "semicolon", "colon", b) any symbol or word without spaces. Default = "tab".
-column = number of the column where the ID's are located. Default=1.
-header = input file header. Options: "yes", "no". Default = "no".

Example:
./REGSCAN -M remseqdup -file myfile.txt **-out** results.txt **-delim** tab **-column** 2 **-header** yes

4.9 “multicount” function

This function counts the occurrences of ID's in a specific column of a file based on a reference list given in another file. In essence, it attaches a number to each entry in the reference list stating how many times this entry was encountered in the main file. It can also break the entries into fragments based on the presence of an additional delimiter when '-iddlim' is selected. For example “waist/hip” is normally considered to be one ID. However, when '-iddelim /' is set it is considered as two separate ID's: “waist” and “hip”.

Prototype: **./REGSCAN -M multicount -file -out -list -delim (-iddelim) -column -header**

-file = file name. REQUIRED.
-out = output file name. Default = input file name + ".out".
-list = this file contains the reference ID's, one per row. REQUIRED.
-delim = file delimiter. Options: a) "tab", "space", "comma", "semicolon", "colon", b) any symbol or word without spaces. Default = "tab".
-iddelim = additional delimiter used to fragment the ID's in the main file. Deafult = not used.
-column = number of the column where the ID's are located. Default=1.
-header = input file header. Options: "yes", "no". Default = "no".

Examples:
./REGSCAN -M multicount -file myfile.txt **-out** results.txt **-list** references.txt **-delim** tab **-column** 2

-header yes

When the entries from column 2 of myfile.txt are compared against the references in references.txt they are treated “as is” (without any modifications).

./REGSCAN -M multicount -file myfile.txt **-out** results.txt **-list** references.txt **-delim** tab **-iddelim** / **-column** 2 **-header** yes

When the entries from column 2 of myfile.txt are compared against the references in references.txt they are first fragmented into smaller IDs wherever “/” is encountered. For example “waist/hip” is treated as two separate ID's: “waist” and “hip”.

5.0 “combifilter” function

This function is used for filtering data from regression analysis involving combinatorial traits. It extracts the trait combinations that are “better” hits (as judged by a statistic such as p-value) than the corresponding single traits alone. The threshold value for “better” can be changed, the direction for “better” can be changed. This function reports whether the combinatorial trait was “better” than both of the single traits or only one of them.

Prototype: **./REGSCAN -M combifilter -file -out -delim -iddelim -column -valuecolumn – markercolumn -header -dir -threshold -factor -missing -missing-ok**

-file = file name. REQUIRED.
-out = output file name. Default = input file name + ".out".
-delim = file delimiter. Options: a) "tab", "space", "comma", "semicolon", "colon", b) any symbol or word without spaces. Default = "tab".
-iddelim = additional delimiter used in combinatorial traits. Deafult = not used. Example: “/”.
-column = number of the column where the trait names are located. Default=2.
-valuecolumn = number of the column where the statistical values are located. Default=12.
-markercolumn = number of the column where the marker names are located. Default=1.
-header = input file header. Options: "yes", "no". Default = "yes".
-dir = direction for deciding which of the two values is better when comparing. Options: “smaller”, “larger”. Default = “smaller. “Smaller” means that the smaller of the two values is considered better, “larger” means that larger of the two values is considered better.
-threshold = the value limit for value in column defined by '-valuecolumn'. If direction ('-dir') is “smaller” then the threshold value is the upper limit for the combinatorial trait to be reported, if it is “larger” then the threshold value is the lower limit for the combinatorial trait to be reported.
-factor = the factor by which one value has to be better than the reference value. See examples below.
-missing = used to indicate the missing values. Options: any symbol or word without spaces. Default = "NA".
-missing-ok = are the missing values treated as positive hits. Options: “yes, “no”. Default: “no”.

Examples:
./REGSCAN -M combifilter -file myfile.txt **-out** results.txt **-delim** tab **-iddelim / -column** 2 **-valuecolumn** 12 **– markercolumn** 1 **-header** yes **-dir** smaller **-threshold** 5e-8 **-factor** 2 **-missing** NA

Here the combinatorial trait separator is "/". The ratios that have values in column 12 that are smaller than 5e-8 AND are two times smaller than at least one of the reference values (for the corresponding single traits) are considered good and are reported. Reference trait value is obtained from the line where the trait was analyzed alone. Note that for this filtration to work, the "-M gwas" settings needed to be relaxed enough to produce sufficient data.

./REGSCAN -M combifilter -file myfile.txt **-out** results.txt **-delim** tab **-iddelim / -column** 2 **-valuecolumn** 10 – **markercolumn** 1 **-header** yes **-dir** larger **-threshold** 1 **-factor** 1.5 **-missing** NA **-missing-ok** yes

Now the combinatorial trait values need to be equal to or larger than 1 AND 1.5 times larger than at least one of the reference value (OR at least one reference value must be missing (due to '-missing-ok yes')) for them to be reported.

The output file contains the following columns:
Marker: marker name
Trait1: first constituent of the combinatorial trait
Trait2: second constituent of the combinatorial trait
Combination: value (such as p-value) of the combinatorial trait
Trait1: value of the first constituent trait when tested alone
Trait2: value of the second constituent trait when tested alone
OK: whether the combinatorial trait was better than one or both of the constituent traits (when tested alone)

5.1 "multifilter" function

This function performs additional filtering of the data output by the '-M gwas' function. It allows filtering based on multiple (unlimited) parameters, using both the AND and OR logical operators. It can also deal with missing values by either considering them just as values or ignoring them.

Prototype: **./REGSCAN -M multifilter -file -out -delim -missing -ignore-missing -logic -dir -header -filter**

-file = file name. REQUIRED.
-out = output file name. Default = input file name + ".out".
-delim = file delimiter. Options: a) "tab", "space", "comma", "semicolon", "colon", b) any symbol or word without spaces. Default = "tab".
-missing = used to indicate the missing values. Options: a) any symbol or word without spaces, b) "none". Default = "NA".
-ignore-missing = whether missing values (defined by '-missing') are ignored (always treated as OK) or not (treated as defined bu the user, see below). Options: "yes", "no". Default: "no".
-logic = defines logical operator used for filtering. Options: "and", "or", Default: "and".
-dir = whether to include or exclude entries that met the criteria defined by '-logic' and '-filter'. Options: "include, "exclude". Default: "include".
-header = input file header. Options: "yes", "no". Default = "yes".

-filter = the main parameter that defines how to filter. The argument is composed of values and letters separated by "_" and ",". Each filter has the following structure: "name_parameter_value"; different filters are separated by ",". Name = what column to consider (this is an integer when header is not present or name when header is present). If the absolute value of the parameter is used, the name is preceded with "abs:" (see example below)). Parameter = how to treat the values (options: "s"=smaller than, "se" or "es" = smaller than or equal to, "l" means larger than, "le" or "el" means larger than or equal to, "e" = equal, "ne"or "en" means not equal). Parameter values are case-insensitive. Value = the reference value against which all entries are compared.

Examples:

./REGSCAN -M multifilter -file mydata.txt **-out** results.txt **-delim** tab **-missing** NA **-ignore-missing** yes **-logic** and **-dir** include **-header** yes **-filter** abs:Slope_l_0.01,P_se_5e-8

Data file has a header with column names. "NA" is the missing value and it is ignored (it passes all tests). All entries are included that that have their absolute "slope" value larger than 0.001 AND their "P" value smaller than or equal to 5e-8.

./REGSCAN -M multifilter -file mydata.txt **-out** results.txt **-delim** tab **-missing** NA **-ignore-missing** yes **-logic** or **-dir** exclude **-header** yes **-filter** SEslope_le_1,P_l_5e-8

Data file has a header with column names. "NA" is the missing values and it is ignored (it passes all tests). All entries are excluded that that have their SEslope value larger than or equal to 1 OR their "P" value larger than 5e-8.

./REGSCAN -M multifilter -file mydata.txt **-out** results.txt **-delim** tab **-missing** NA **-ignore-missing** yes **-logic** or **-dir** include **-header** yes **-filter** Slope_l_0.5,Slope_s_-0.5

Data file has a header with column names. "NA" is the missing values and it is ignored (it passes all tests). All entries are included that that have their slope value over 0.5 OR under -0.5. Note that each column can have more than one filter set upon them.

./REGSCAN -M multifilter -file mydata.txt **-out** results.txt **-delim** tab **-missing** NA **-ignore-missing** yes **-logic** and **-dir** exclude **-header** no **-filter** abs:1_e_2,2_ne_5,2_l_2

Data table does not have a header hence the column numbers must be used as identifiers instead of their names. "NA" is the missing values and it is ignored (it passes all tests). All entries are excluded that have their absolute value of column 1 equal to 2 AND the value in column 2 not equal to 5 AND the value in column 2 larger than 2.

./REGSCAN -M multifilter -file mydata.txt **-out** results.txt **-delim** tab **-missing** NA **-ignore-missing** no **-logic** or **-dir** include **-header** no **-filter** 2_l_3,2_ne_NA

Data table does not have a header hence the column numbers must be used as identifiers instead of their names. "NA" is not ignored, it is treated as any other value. Here only the entries are included that have a value larger than 3 in column 2 OR don't have "NA" in column 2.

5.2 “extractid” function

This function allows to extract rows from a file that has certain string or a part of that string in the user-defined column(s).

Prototype: **./REGSCAN -M extractid -file -out -delim -header -column -is -contains -dir**

-file = file name. REQUIRED.
-out = output file name. Default = input file name + ".out".
-delim = file delimiter. Options: a) "tab", "space", "comma", "semicolon", "colon", b) any symbol or word without spaces. Default = "tab".
-header = input file header. Options: "yes", "no". Default = "no".
-column = number of the column that is searched. Options: a) any integer smaller than the number of columns, b) 0. Zero (0) means that all columns are searched. Default = “0”.
-is = reference string compared to the string located in the selected column. Only FULL matches are returned as positive.
-contains = reference string compared to the string located in the selected column. Both FULL and PARTIAL matches are returned as positive. Note: When '-is' and '-contains' are both defined, only '-is' is used.
-dir = whether to include or exclude entries that met the criteria defined by '-is' or '-contain'. Options: “include, “exclude”. Default: “include”.

Examples:
./REGSCAN -M extractid -file mydata.txt **-out** results.txt **-delim** space **-header** yes **-column** 0 **-contains** protein **-dir** exclude

All columns are searched for entries containing the word “protein”. The rows that do not contain it as part of the string are written into the output file.

./REGSCAN -M extractid -file mydata.txt **-out** results.txt **-delim** comma **-header no -column** 2 **-is** NA -**dir** include

Column 2 is searched. If this column contains “NA” (exact match) then the corresponding row is written into the output file.

V. WORKED EXAMPLE

Below is a step by step worked example for the most typical RegScan usage – analyzing trait ratios. Please find the example files “TEST.impute” , “TEST.sample” and “references.txt”. Please follow these exercises in the correct order.

Here's the general work flow:

STEP 1

1. Convert the sample file to trait ratios file usable by RegScan.

a) Remove the oops line and the extra info from the columns.

TEST.sample contains phenotype names in the file header (row 1) and phenotype data in rows 3-44. The phenotypes are only in columns 4-13. Let's extract the phenotype fields:

./REGSCAN -M extract -file TEST.sample **-out** phenotypes.txt **-rows** 1,3-44 **-columns** 4-13

Note: *Continuous row or column ranges are specified with “-” whereas individual rows or columns are separated by comma. This example of 1,3-44 means: 1 and 3 through 44. If delimiter is not space, the correct delimiter should be declared using the '-delim' switch (examples: -delim space, -delim comma).*

b) Create the trait ratios file. Lets assume that we want trait ratios rather than simple traits.

./REGSCAN -M combitable -file phenotypes.txt **-out** combinations.txt **-header** yes **-columns** 1-10 **-function** divide

Note: *Column or row max value does not need to match the end of file. If the range exceeds the end of file, it it taken to equal the end of file. It is important to define header to have the trait ratio names derived logically from the original names.*

This function adds combinatorial traits to the original ones. If you want to use the combinations only, they should be extracted from combinations.txt using the “extract” function as shown above.

Typically these results would be adjusted for covariates and inverse normally transformed (see above). For the sake of learning how RegScan works we are skipping this step.

c) Transpose the trait ratios file to make RegScan style trait file

./REGSCAN -M transpose -file combinations.txt **-out** TEST.regscan **-col-chunk** 100

Note: *The '-col-chunk' switch merely defines how many columns are handled together. It has no effect here but this can make a difference with the speed of transforming very large data files.*

2. Perform linear regression analysis:

./REGSCAN -M gwas -gfile TEST.impute **-pfile** TEST.regscan **-out** RESULTS.txt **-statistic** p **-statlimit** 5e-2 **-slope** 0.0 **-selimit** 1e3 **-maclimit** 1 **-summary** yes

3. Find out how many times each marker was considered interesting

./REGSCAN -M remseqdup -file RESULTS.txt **-out** markercounts.txt **-delim** tab **-header** yes

4. Find out how many marker hits each trait ratio had

Count full IDs:
./REGSCAN -M multicount -file RESULTS.txt **-out** traitcounts1.txt **-list** references.txt **-delim** tab **-column** 2 **-header** yes

Count ID fragments:
./REGSCAN -M multicount -file RESULTS.txt **-out** traitcounts2.txt **-list** references.txt **-delim** tab **-iddelim** / **-column** 2 **-header** yes

Note: *The file "references.txt" was included in your download package.*

STEP 2

5. Use "combifilter" function to see if any of the trait ratios had a lower p-value than those of its constituents

For this analysis to work we need to have more data than we generated in step 2. Let's re-run this test with more relaxed thresholds:

./REGSCAN -M gwas -gfile TEST.impute **-pfile** TEST.regscan **-out** RESULTS2.txt **-statistic** p **-statlimit** 1 **-slope** 0.0 **-maclimit** 1

Next isolate the promising ratios:

./REGSCAN -M combifilter -file RESULTS2.txt **-out** ratio_hits.txt **-delim** tab **-iddelim** / **-column** 2 **-valuecolumn** 12 **– markercolumn** 1 **-header** yes **-dir** smaller **-threshold** 5e-2 **-factor** 10

Several trait ratios had their p-value at least 10 times lower than at least one of the constituent traits. Note that two deletions stand out among other markers.

6. Use "multifilter" function to compress the RESULTS2.txt file from "combifilter" function

./REGSCAN -M multifilter -file RESULTS2.txt **-out** filtered_hits.txt **-delim** tab **-logic** and **-dir** include **-header** yes **-filter** abs:Slope_1_0.05,P_se_0.2

This compressed the original results and eliminated the "least interesting results". We only retained the entries that had absolute slope over 0.05 and P value smaller than or equal to 0.2.

Now, let's redo the "combifilter" analysis:

./REGSCAN -M combifilter -file filtered_hits.txt **-out** ratio_hits2.txt **-delim** tab **-iddelim** / **-column** 2 **-valuecolumn** 12 **– markercolumn** 1 **-header** yes **-dir** smaller **-threshold** 5e-2 **-factor** 10

Note that the hits list (ratio_hits2.txt) is now shorter than previously (ratio_hits.txt). Note: You may also add '-missing-ok yes' to include the ratio hits that had at least one of the single trait hits missing.

7. Use "extractid" to isolate only one trait from the results.

The "gwas" function deposits all results in one file (RESULTS2.txt). The user may be interested in seeing the data for just one trait. Let's extract data for all entries involving "pheno7":

./REGSCAN -M extractid -file RESULTS2.txt **-out** pheno7involved.txt **-delim** tab **-header** yes **-column** 2 **-contains** pheno7 **-dir** include

Here we extract all entries that are for "pheno7" only (no combinatorial traits):

./REGSCAN -M extractid -file RESULTS2.txt **-out** pheno7only.txt **-delim** tab **-header** yes **-column** 2 **-is** pheno7

Here we extract only single traits (no ratios):

./REGSCAN -M extractid -file RESULTS2.txt **-out** single_tarits_only.txt **-delim** tab **-header** yes **-column** 2 **-contains** / **-dir** exclude

Thank you for reading the RegScan user guide! We are looking forward to your feedback.
